# Actinomycosis in a Gallbladder Specimen: A Case Report

**DOI:** 10.7759/cureus.77050

**Published:** 2025-01-06

**Authors:** Rahul R Mor, Hiba Shanti

**Affiliations:** 1 Minimal Access Surgery, King's College Hospital NHS Foundation Trust, London, GBR

**Keywords:** actinomycosis, acute cholangitis, endoscopic retrograde cholangiopancreatography, gallbladder, gallstones, laparoscopic cholecystectomy

## Abstract

Actinomycosis is a chronic, granulomatous infection caused by *Actinomyces* species, a group of anaerobic, gram-positive bacteria commonly found in the human oral cavity, gastrointestinal, and female genital tracts. Although it predominantly affects the cervicofacial region, rare manifestations such as gallbladder actinomycosis can occur. This report presents a case of gallbladder actinomycosis in a 61-year-old man who presented with a two-week history of right upper quadrant pain, jaundice, nausea, and vomiting. Imaging revealed biliary obstruction with common bile duct stones, leading to endoscopic retrograde cholangiopancreatography and subsequent laparoscopic cholecystectomy. Histopathological examination identified *Actinomyces* species, confirmed by Gram, PAS, and Grocott staining. While prolonged antibiotic therapy is the cornerstone of treatment, localized infections with complete surgical excision may not necessitate extended antibiotic use, as highlighted in this case.

## Introduction

Actinomycosis is a chronic, granulomatous infection caused by *Actinomyces*, a group of filamentous, gram-positive, non-acid, fast anaerobic bacteria commonly found in the oral cavity, gastrointestinal tract, and female genital tract. While traditionally associated with the cervicofacial region, *Actinomyces *can infect various other organs [1].

Gallbladder actinomycosis is rare, with hepatobiliary actinomycosis accounting for approximately 5% of all abdominal cases of actinomycosis [2-4]. Due to its rarity, particularly in the gallbladder, the precise incidence remains difficult to determine. Previous reports suggest that gallbladder actinomycosis represents less than 1% of all actinomycosis cases [5-7]. It frequently appears as a mass, an abscess, or presents with obstructive jaundice - mimicking more common gallbladder conditions such as gallstones or malignancies. This case report describes a rare instance of gallbladder actinomycosis.

## Case presentation

A 61-year-old man presented with a two-week history of right upper quadrant pain and jaundice, accompanied by nausea and vomiting. He denied prior similar episodes, fever, or weight loss. His past medical history included a right inguinal hernia but no diabetes and no causes for immunosuppression. He was not on any regular medications.

On examination, the patient appeared jaundiced and had right upper quadrant tenderness with a positive Murphy’s sign. Laboratory tests revealed a white cell count of 15 × 10⁹/L (2.9-9.6 10⁹/L), C-reactive protein at 229 mg/L (<5 mg/L), total bilirubin at 522 µmol/L (<21 µmol/L), aspartate aminotransferase at 85 U/L (10-50 U/L), and alkaline phosphatase at 348 U/L (30-130 U/L). A computed tomography (CT) scan showed intrahepatic and extrahepatic biliary dilatation, with multiple stones in the common bile duct (CBD). The patient presented with Charcot's triad - fever, right upper quadrant tenderness, and jaundice - highly suggestive of acute cholangitis. The diagnosis was supported by elevated inflammatory markers, bilirubin, and alkaline phosphatase levels, with a CT scan confirming the diagnosis and choledocholithiasis as the underlying cause of obstruction.

The patient was started on an intravenous antibiotic, tazocin (piperacillin-tazobactam), and underwent endoscopic retrograde cholangiopancreatography (ERCP), during which sphincterotomy was performed, pus was drained, and stones were extracted. However, the CBD could not be fully cleared, and a plastic stent was placed. His symptoms improved, and he was discharged after four days with co-amoxiclav (amoxicillin/clavulanic acid) to complete a total of 10 days of antibiotics. A follow-up ERCP was performed, achieving complete clearance of the CBD.

The patient subsequently underwent laparoscopic cholecystectomy. Intraoperatively, the gallbladder was found to be thickened with dense fibrosis and adherent to the liver but not infiltrating it. A cholangiogram through the cystic duct showed no CBD stones. The patient recovered well and was discharged the following morning.

**Figure 1:**
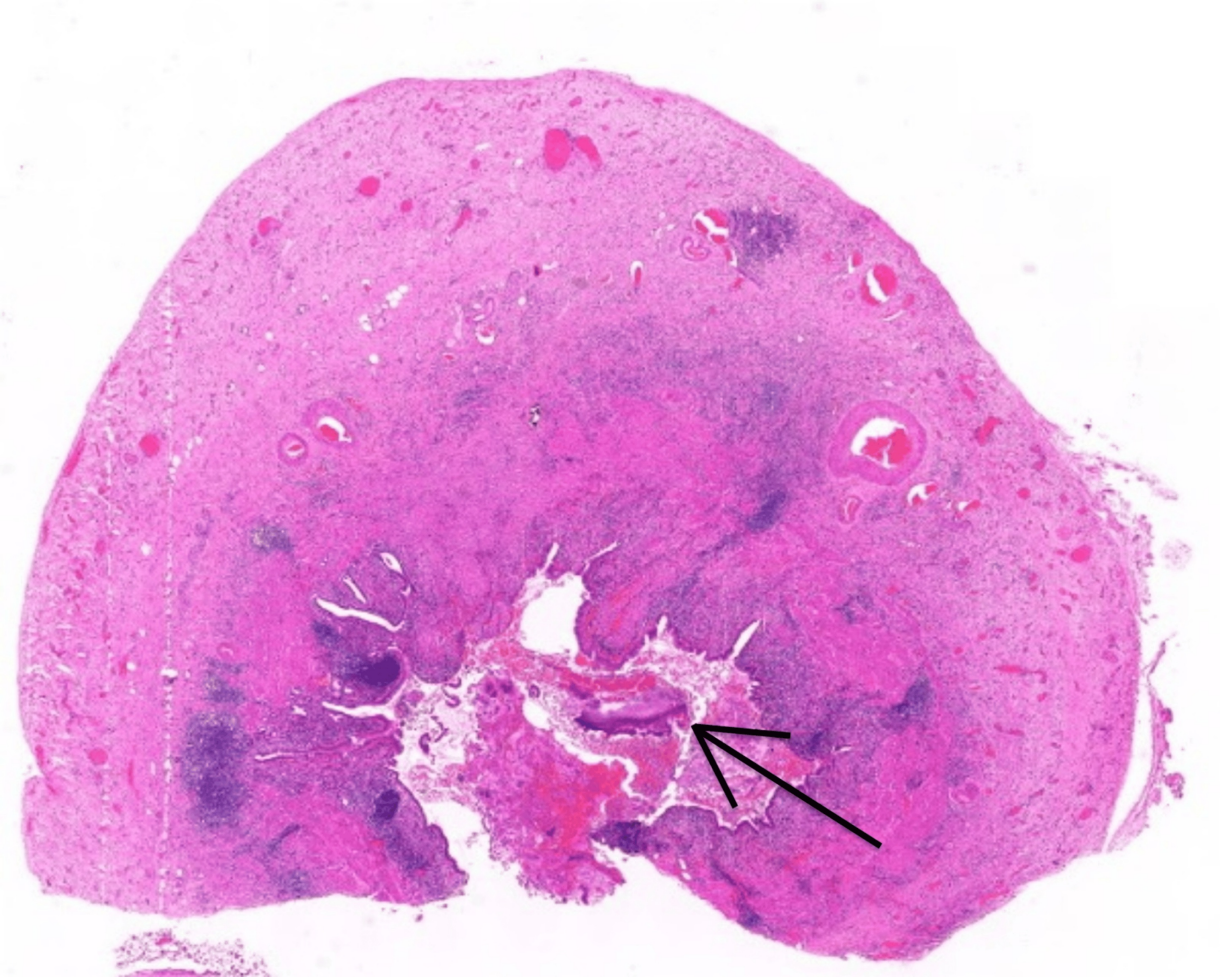
Histopathological slide of an inflamed gallbladder with an intraluminal cluster of organisms (hematoxylin and eosin stain, magnification x1.3)

**Figure 2:**
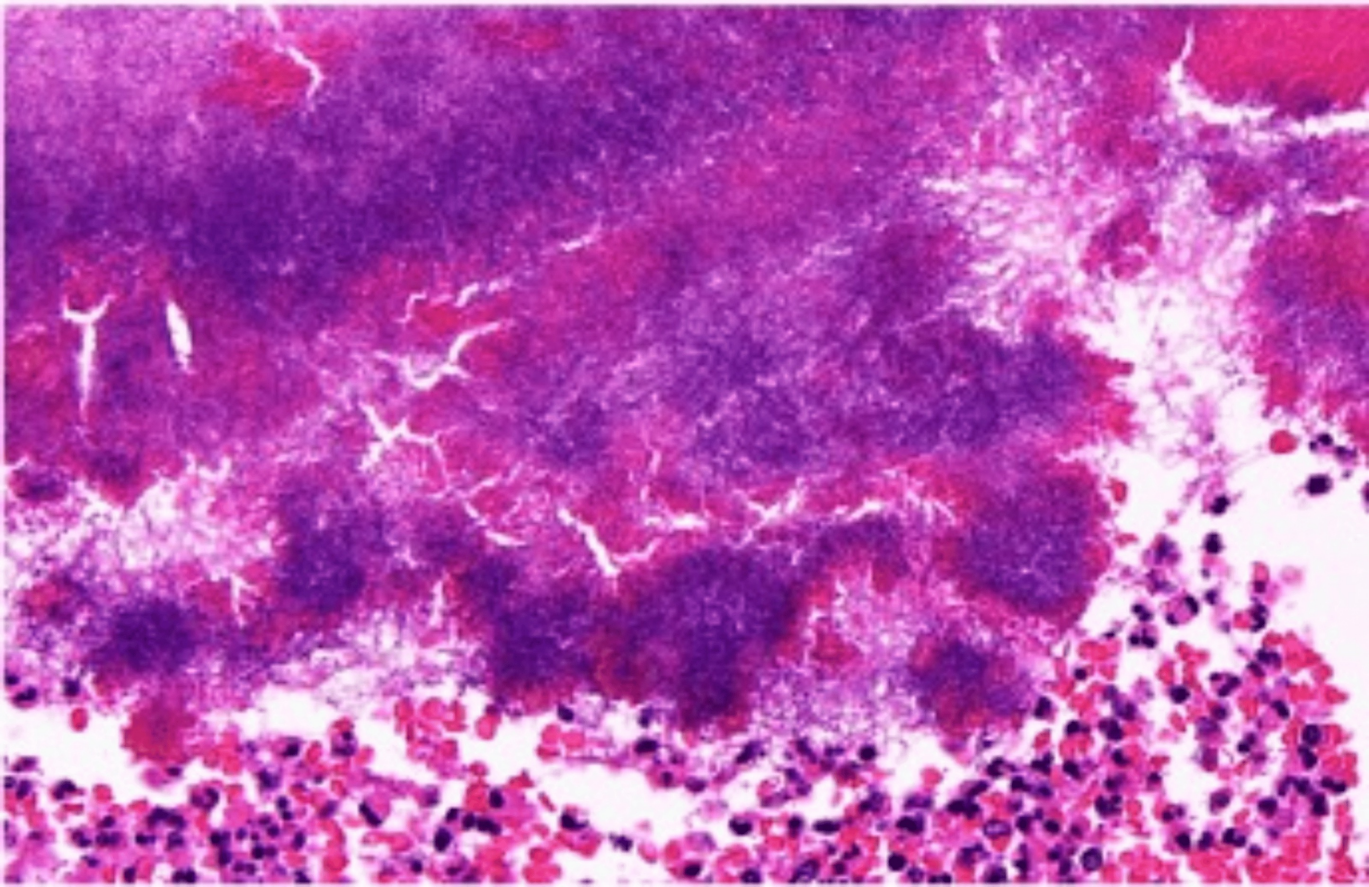
Magnified image of the cluster of organisms (hematoxylin and eosin stain, magnification x10)

**Figure 3:**
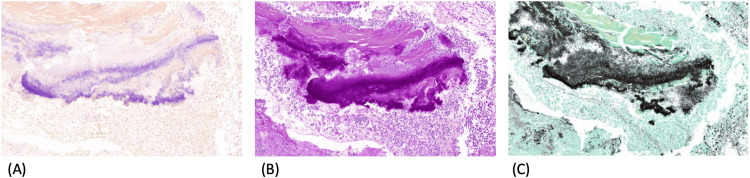
Positive stains of Actinomyces for Gram (A), PAS (B), and Grocott (C). All three slides have a magnification of x10 PAS: periodic acid-Schiff

Routine histopathological examination revealed a large cholesterol lithiasis, 25 mm, with an erosion of the mucosa and a thickened fibrous gallbladder wall, with no additional macroscopic findings (sulfur granules were not present). Histologically, a moderate degree of chronic cholecystitis was observed with focal activity and suppurative inflammation. Notably, a single intraluminal cluster of microorganisms with features consistent with *Actinomyces *was identified (Figures 1-2). The organism stained positive for Gram, Periodic Acid-Schiff (PAS), and Grocott (Figures 3A-3C). There was no evidence of dysplasia or malignancy.

The case was discussed in the microbiology multidisciplinary team (MDT) meeting. Given that the patient remained asymptomatic, lacked abscess formation, and had undergone complete gallbladder resection, it was concluded that there was no indication for a prolonged course of antibiotics, as there was no clinical syndrome consistent with actinomycosis.

## Discussion

Actinomycosis is a chronic, granulomatous infectious disease caused by *Actinomyces *species, a group of anaerobic, Gram-positive bacteria that are part of the normal flora of the human mouth, gastrointestinal tract, and female genital tract [1,2]. These bacteria typically inhabit mucosal surfaces and do not cause disease unless there is a disruption in the normal barrier, such as trauma, surgery, or immunosuppression [1,4,6,8].

When infection occurs, *Actinomyces *can invade surrounding tissues, forming abscesses, fibrotic scars, and the characteristic sulfur granules, which are yellowish, grain-like aggregates of bacteria surrounded by inflammatory cells [1,4,9]. Actinomycosis is most commonly seen in the cervicofacial region, followed by the abdominal and pelvic areas. Abdominal actinomycosis accounts for around 20% of presentations [2,4].

Gallbladder actinomycosis is an exceedingly rare manifestation, representing only a small fraction of abdominal actinomycosis cases. Most reported cases are associated with stones in the gallbladder, bile duct, malignancy, or immunosuppression [1,4,6]. Studies indicate that the average age of presentation is around 65 years, and while some studies showed a prevalence in females, others did not show a difference [6,7].

The pathogenesis of gallbladder actinomycosis starts with bacterial spread to the gallbladder. While hematogenous spread to the liver has also been reported, lymphatic spread is not believed to occur in actinomycosis due to the large size of the filaments [6,8]. In this case, we proposed that ERCP provided a direct pathway from the gastrointestinal tract, and sphincterotomy facilitated bacterial reflux from the duodenum [3-6,10].

Biliary obstruction, particularly in the presence of gallstones, establishes an anaerobic environment conducive to bacterial proliferation. The infection typically progresses slowly, resulting in the formation of abscesses, fibrosis, and adhesions, which may extend to adjacent structures such as the liver and duodenum [1]. *Actinomyces *generally do not grow in the presence of bile salts, except for certain strains of *Actinomyces naeslundii*. Consequently, the presence of sulfur granules in the gallbladder is regarded as indicative of invasive disease rather than simple colonization [6,7].

The infection typically presents with nonspecific symptoms, including right upper quadrant pain, fever, and jaundice, which can resemble other conditions like biliary infections or malignancies [1]. There are no specific characteristics of imaging. However, features like multiple abscesses and/or enterocutaneous fistulas can be seen, particularly in chronic infections [1,4,9].

Diagnosis is challenging and is confirmed through tissue biopsy and/or anaerobic culture of pus, with sulfur granules and gram-positive bacteria being common findings [1,2,4]. However, these features are present in only 75% of cases [1,3,9].

Intraoperative findings typically include dense adhesions, fibrosis, and a mass infiltrating adjacent organs, along with the presence of abscesses or fistulas [11-13]. Laparoscopic cholecystectomy is generally feasible; however, the conversion rate to open surgery is higher in gallbladder actinomycosis due to the presence of fibrosis, abscesses, involvement of adjacent organs, or suspicion of malignancy [11].

The standard treatment for actinomycosis typically involves prolonged antibiotic therapy, with penicillin being the most commonly used agent. Although the prognosis is generally favorable, chronic infections complicated by abscesses or fistulas can lead to significant morbidity [1]. To avoid relapse, prolonged treatment is advisable, often administered for 6-12 months. However, there are some reports of successful short-term treatment with antibiotics where abscesses were cleared surgically. If short-term antibiotic treatment is attempted, the clinical and radiological response should be closely monitored [9,14].

## Conclusions

Gallbladder actinomycosis is a rare but important differential diagnosis in patients presenting with atypical biliary symptoms. This case highlights the importance of histopathological examination in achieving an accurate diagnosis. However, in our patient, this finding is most likely incidental. In cases where the infection is localized and the affected organ is completely excised, as demonstrated in this case, prolonged antibiotic therapy of 6-12 months may be unnecessary.
